# Targeted Sorting of Single Virus-Infected Cells of the Coccolithophore *Emiliania huxleyi*

**DOI:** 10.1371/journal.pone.0022520

**Published:** 2011-07-26

**Authors:** Joaquín Martínez Martínez, Nicole J. Poulton, Ramunas Stepanauskas, Michael E. Sieracki, William H. Wilson

**Affiliations:** Bigelow Laboratory for Ocean Sciences, West Boothbay Harbor, Maine, United States of America; Mt. Alison University, Canada

## Abstract

Discriminating infected from healthy cells is the first step to understanding the mechanisms and ecological implications of viral infection. We have developed a method for detecting, sorting, and performing molecular analysis of individual, infected cells of the important microalga *Emiliania huxleyi,* based on known physiological responses to viral infection. Of three fluorescent dyes tested, FM 1-43 (for detecting membrane blebbing) gave the most unequivocal and earliest separation of cells. Furthermore, we were able to amplify the genomes of single infected cells using Multiple Displacement Amplification. This novel method to reliably discriminate infected from healthy cells in cultures will allow researchers to answer numerous questions regarding the mechanisms and implications of viral infection of *E. huxleyi*. The method may be transferable to other virus-host systems.

## Introduction

Viral infection greatly influences the biogeochemistry and genetic variability that sustain marine phytoplankton communities by accelerating the lysis of bacteria and phytoplankton and serving as vectors for horizontal gene transfer [1]. However, the inability to separately investigate infected and healthy phytoplankton cells in the environment limits our understanding of the ecological and biogeochemical implications of viral infection. Subtle changes in infected cells of a particular phytoplankton species are almost impossible to detect from bulk measurements using standard mass filtering [2] and subsequent biogeochemical or molecular analysis. This is particularly true when the species of interest is not dominant and/or when infected cells only represent a small fraction of the community. These limitations evidence the need for a method that allows discriminating and isolating infected phytoplankton cells from environmental samples.

Previous studies have shown that viral infection of the marine microalga *Emiliania huxleyi* leads to intracellular accumulation of reactive oxygen species (ROS) [3] and plasma membrane patchiness (blebbing) due to increased production of a dense lipid excreted to the cell surface [4]. Here, we took advantage of these physiological responses to develop a method for distinguishing single infected cells within a phytoplankton culture using flow cytometry prior to high throughput physical separation, whole genome amplification and molecular analysis. We chose the *E. huxleyi* host-virus system because of its wide distribution, high abundance and importance in the ocean's biogeochemistry [5], [6], as well as the ease with which the host can be grown and the virus propagated in the laboratory. Additionally, recurring vast *E. huxleyi* blooms have been reported to be terminated by viral infection [7], [8], [9] and extensive sequence information is available for *E. huxleyi* strain CCMP 1516 (http://genome.jgi-psf.org/Emihu1/Emihu1.home.html) and *E. huxleyi*-specific virus strain EhV-86 [10], facilitating molecular analysis.

We tested and compared three flow cytometric assays for the discrimination of healthy and infected cells: 1) lipid dye FM 1-43 for detection of membrane blebbing [4]; 2) CM-H_2_DCFDA for detection of accumulated intracellular ROS [3]; 3) DNA dye SYBR Green I for detection of increased total DNA in infected cells as virus progeny accumulates intracellularly prior to lysis (as reported for *Chlorella* NC64A [11]). We then judged the suitability of the sorted cells for whole genome multiple displacement amplification (MDA) to generate sufficient good quality genomic DNA for downstream molecular analysis. MDA amplicons were screened by PCR using generic and specific primers for *E. huxleyi* and *E. huxleyi*-specific viruses (EhVs) respectively.

## Results

**Figure 1:**
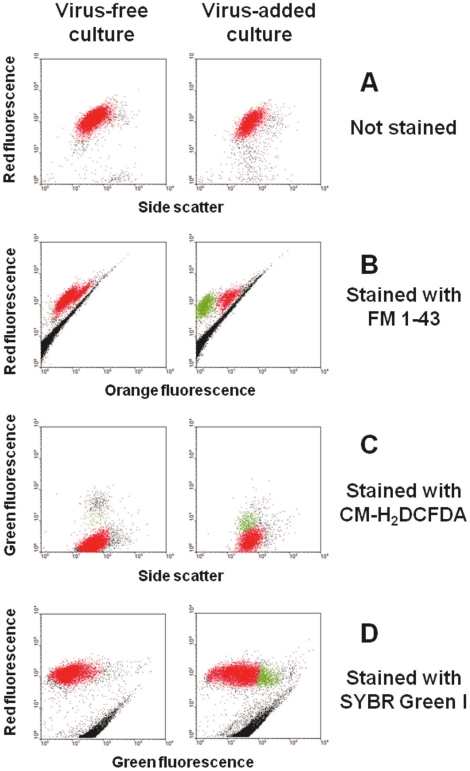
Representative flow cytometry plots showing *E. huxleyi* cells, inoculated and non-inoculated with viruses (20 hours PI) (A) without fluorescence dye, or stained with the fluorescence dyes (B) lipid-specific dye FM 1-43, (C) CM-H_2_DCFDA for detection of accumulated Reactive Oxygen Species in cells and (D) DNA dye SYBR Green I. Infected and non-infected cells were discriminated on the basis of their red autofluorescence (610 nm) or the green fluorescence (522 nm) of SYBR Green I and CM-H_2_DCFDA versus side scatter, green dye fluorescence or orange lipid dye FM 1-43 fluorescence (488 nm).

In an initial experiment we compared the efficacy of three fluorescence probes for discriminating infected from healthy *E. huxleyi* strain CCMP 1516 cells at different times during the infection process (2 h, 6 h, 20 h, 24 h, 42 h and 48 h post-inoculation, PI). At each PI time point three aliquots from both a virus-free and a virus-added culture were each labeled with either fluorescent dye FM 1-43, CM-H_2_DCFDA or SYBR Green I. A fourth aliquot received no stain. In the virus-free aliquots all the cells were uniformly labeled and appeared as a single group (**Fig. 1**). FM 1-43 was found to be the most suitable dye for our host-virus system, allowing the most distinctive discrimination between cell subpopulations in the virus-added culture (one subpopulation with high-orange fluorescence as that in the virus-free culture and a second with low-orange fluorescence) compared to CM-H_2_DCFDA and SYBR Green I (**Figs. 1B-D** **).** Furthermore, distinctive subpopulations were resolved with FM 1-43 from 6 hours PI while the other two fluorescent dyes distinguished cell subpopulations (based on acquired green fluorescence) only from 20 hours PI (**Figs. S1, S2, S3**). In the absence of fluorescence dyes the cells' red autofluorescence and low green fluorescence levels did not change throughout the 48 h sampling period both in the virus-free and the virus-added cultures (**Fig. S4**).

In an independent experiment analogous to the previous one, we mixed FM 1-43 dye-labeled aliquots from virus-free and virus-added *E. huxleyi* cultures (1∶1 ratio) at 20 h PI and sorted 84 single cells with high- and 84 single cells with low-orange fluorescence (red and green clusters, respectively, in **Fig. 1B**) into a 384-well plate. Additionally, we included 22 blank wells (no-drop deposition) and 1 positive sorting control well (50 cells in a single well) from each cell subpopulation. After sorting, MDA and real-time PCR screening with both MCP [12] and GPA [13] primers (to detect virus and host respectively) were performed on each well.

Close to 100% of the MDA reactions in wells containing single cells were operationally-defined successful, i.e. critical amplification point (Cp), the time necessary to reach half of the maximum accumulated fluorescence for each sample, was less than 12 hours. In general, low-orange fluorescence cells were MDA-amplified faster than high-orange fluorescence cells (**Table 1** and **Fig. S5**). Cp mean for multiple-cell wells was approximately 4∶30 hours and Cp mean for blank wells was 14∶35 hours, indicating the overall effectiveness of the sorting process, the suitability of the MDA reaction conditions and the lack of DNA contamination in the blanks and the MDA reagents (**Fig. S5**).

**Table 1:** Summary of MDA and PCR results (with MCP and GPA primers, for virus and host respectively) for single sorted cells with addition of fluorescence dye FM 1-43.

Sorted population	MDA^a^		PCR^b^			
			MCP		GPA	
	Cp<8	12>Cp≥8	Cp<8	12>Cp≥8	Cp<8	12>Cp≥8
High-orange fluorescence	62%	36%	17%	3%	36%	70%
Low-orange fluorescence	89%	8%	97%	0%	24%	50%

MDA reactions with Cp>12 hours did not yield any PCR products and therefore are not included in this table.

a Results are presented as percentage of wells containing single-sorted cells. Time for MDA amplification was determined from the critical amplification point (Cp, in hours).

b Results are presented as percentage of positive PCR reactions for each group of MDA reactions, i.e. Cp<8 or 12>Cp≥8.

MCP and GPA PCR reactions yielded amplicons for the multiple-cell wells and did not yield products for any of the blank wells or the few MDA reactions with Cp≥12 hours (data not shown), indicating the suitability of the reaction and the lack of contamination across wells. Ninety seven percent of the MDA products from single sorts with Cp<8 hours from the low-orange fluorescence cells yielded an EhV-MCP amplicon indicating that those single sorted cells were indeed infected with EhV-86. In contrast, we could only amplify the MCP fragment from approximately 17% of MDA amplicons with Cp<8 hours from high-orange fluorescence cells (**Table 1**). *E. huxleyi*-specific GPA reactions were overall more successful on high-orange fluorescence cells compared to infected cells and on MDA products with Cp>8 hours (**Table 1**).

Although FM 1-43 dye was chosen in preference to CM-H_2_DCFDA and SYBR Green I dyes for our host-virus system, we also proved in a separate test that MDA amplification and PCR efficiencies did not depend on whether the cells had been dye-labeled prior to sorting or on the type of dye utilized (**File S1**).

## Discussion

The method we describe here allows the unequivocal identification, isolation, and whole genome amplification of single *E. huxleyi* infected cells from a culture. A sensible starting point when developing a new technique is to investigate manageable components of the oceanic microbial ecosystem, ideally those that are quantitatively significant; hence, our choice of *E. huxleyi*. Yet, it is possible that this method can be used on a range of microalgae or other host species infected by viruses.

Certain changes in the host physiology can be used as indicators of viral infection. For instance, decreased photosynthetic efficiency of infected hosts has been measured as changes in fluorescence quenching [14], [15], [16]. The decline of autofluorescence can sometimes be detected and quantified by flow cytometry but it is not always evident, especially at early stages of infection. Our results show that in the absence of fluorescent dye, the red autofluorescence of both healthy and infected *E. huxleyi* cells remained undistinguishable for at least the first 24 h of study (**Fig. S4**) evidencing the need for a different approach to discriminate healthy from infected cells at earlier stages of infection when the host's genetic material is still intact [17]. Accumulation of intracellular ROS and membrane and cytoplasmic blebbing are indicators of programmed cell death induced by environmental factors such as excessive ultraviolet radiation, nutrient limitation, oxidative stress or viral infection [18]. Viruses have been suggested to be the ultimate cause of phytoplankton loss for which the death apparatus might be seen as a product of host and virus coevolution, with each trying to control it [19]. In this study we took advantage of those known physiological changes observed in *E. huxleyi* in response to viral infection, and found that membrane blebbing as detected with the fluorescent dye FM 1-43 [4] was most suitable for early discrimination of infected cells.

FM 1-43 revealed two cell populations distinguished by the level of orange fluorescence. Low-orange fluorescence cells, infected with EhV-86, had on average lower MDA Cp values than high-orange fluorescence cells, of which only 20% yielded EhV-MCP amplicons. These amplicons could be due to the presence of non-infectious EhV particles nonspecifically attached to some of the high-orange fluorescence cells or it could be that those high-orange fluorescence cells were indeed infected, but at such early stage of infection that membrane blebbing was not yet evident. Infected *E. huxleyi* cells exhibit plasma membrane patchiness only after approximately 2 h PI [4]. The EhV genome is an easier template than the *E. huxleyi* genome, which with its many repeats, GC-rich regions and extensive secondary structure, is a difficult template for amplification (http://genome.jgi-psf.org/Emihu1/Emihu1.home.html). This likely explains the lower MDA Cp values for low-orange fluorescence (infected) cells compared to high-orange fluorescence cells (mostly non-infected). On the contrary, the higher success of *E. huxleyi*-specific GPA reactions on high-orange, compared to low-orange fluorescence cells (**Table 1**) could be explained by the fact that in infected cells (low orange fluorescence signal): 1) the EhV genomes are preferentially amplified by MDA; 2) most of the host genome has already been degraded for the production of new virions hindering amplification of the GPA fragment [17]. However, this may not be the case for other host-virus systems with different viral infection strategies and different genome composition and structures.

This novel method for targeted sorting of infected *E. huxleyi* cells is a powerful tool that opens a range of new opportunities for the investigation of viral infection of this microalgae and possibly of other host-virus systems. The availability of genomic data from single microalgae infected cells can, for instance, help discern the factors that determine virus specificity and host susceptibility as well as gene transfer and co-evolution by facilitating the search for common shared patterns among the genomes of individual host cells and their viruses. Probably the most important improvement that this method offers is the ability to detect and genetically analyze individual infected cells without the need to maintain the host-virus system in the laboratory. Naturally occurring blooms of *E. huxleyi* are frequently terminated by viral infection [7], [8], [9] and therefore the majority of sorted cells that exhibit membrane blebbing or elevated intracellular ROS or total DNA content are likely to be infected. Moreover, results obtained this way from environmental samples will be more ecologically relevant than those from manipulated laboratory conditions which often do not reflect true ecological conditions.

## Materials and Methods

### Host culture

Non-axenic clonal *Emiliania huxleyi* strain CCMP 1516 (3–5 µm) was obtained from the Provasoli-Guillard Center for the Cultivation of Marine Phytoplankton (CCMP, Maine, USA; http://ccmp.bigelow.org/). The cultures were maintained at 15°C and kept at mid-exponential growth phase (approx. 1–2×10^6^ cells ml^−1^) by periodically transferring 5–10% (v/v) culture in fresh f/2-Si seawater medium [20]. Light (250 µmoL photons m^−2^ s^−1^) was supplied by fluorescence tubes under a light-dark cycle of 16∶8h. Cell concentrations were calculated by flow cytometry as described by Marie et al. [21] using a FACScan flow cytometer (Becton Dickinson, Franklin Lakes, NJ), equipped with an air-cooled laser providing 50 mW at 488 nm and with standard filter set-up. Deionised water was used as sheath fluid.

### Virus pathogen

*E. huxleyi-*virus (EhV) strain EhV-86 [12] was obtained from the Plymouth Virus Collection (UK). Fresh working solutions of EhV-86 lysate were produced prior to performing an experiment. Briefly, 1 ml lysate was added to 50 ml of an exponentially growing culture of *E. huxleyi* strain CCMP 1516. Once clearing of the host culture was observed, the lysate was passed through a 0.2 µm syringe filter (Sartorius AG, Germany) and the filtrate containing virus was stored at 4°C. Virus concentration was calculated by flow cytometry using SYBR Green I as described by Brussaard [22].

### Fluorescent cell labeling

1 ml *E. huxleyi* culture aliquots (concentration was adjusted to approx. 1.4×10^5^ cells ml^∼1^) were placed into microcentrifuge Eppendorf tubes and labeled with either fluorescent dye N-(3-riethylammoniumpropyl)-4-[4-(dibutylamino)styryl] pyridinium dibromide (FM 1-43, Invitrogen Co., Carlsbad, CA, USA), 5-(and-6)-chloromethyl-2′,7′-dichlorodihydrofluorescein diacetate (CM-H_2_DCFDA, Molecular Probes Inc., Eugene, OR, USA) or SYBR Green I (Molecular Probes Inc., Eugene, OR, USA). The cells were incubated with 10 µM (final concentration) FM 1-43 [4], 5 mM (final concentration) CM-H_2_DCFDA [3] or SYBR Green I (5×10^5^ dilution of commercial stock) for 30 min, 60 min or 15 min, respectively, in the dark at 15°C. In an initial experiment, an *E. huxleyi* culture aliquot was incubated for 10 minutes with 100 mM H_2_O_2_ to artificially elevate the intracellular ROS concentration prior to addition of CM-H_2_DCFDA dye, as a control to verify the effectiveness of the CM-H_2_DCFDA labeling. H_2_O_2_ is the most stable of the ROS and is capable of rapid diffusion across cell membranes [23]. Labeled *E. huxleyi* cells were discriminated by flow cytometry on the basis of their red autofluorescence at 610 nm versus side scatter, the green fluorescence of the CM-H2DCFDA and SYBR Green I dyes at 522 nm or the orange fluorescence of the FM 1-43 dye at 488 nm, accordingly.

### Fluorescence-activated cell sorting

Prior to cell sorting, samples were diluted 10-fold with sterile-filtered seawater and pre-screened through a 70 um mesh-size cell strainer (BD). Sorting was done with a MoFlo™ (Beckman Coulter) flow cytometer using a 488 nm argon laser for excitation, a 70 µm nozzle orifice and a CyClone™ robotic arm for droplet deposition into microplates. The “single 1 drop” mode was used for maximal sort purity, which ensures the absence of non-target particles within the target cell drop and the drops immediately surrounding the cell.Extreme care was taken to prevent sample contamination by any non-target DNA. Instruments and reagents were decontaminated as previously described [24]. Cell sorting was performed in a HEPA-filtered environment. The cytometer was triggered on side scatter, the sort gates were based on red autofluorescence and side scatter for not-labelled cultures, on red autofluorescence versus orange fluorescence for FM 1-43-labelled cells, on red autofluorescence or green fluorescence versus side scatter for CM-H_2_DCFDA-labelled and on red autofluorescence versus side scatter or green fluorescence for SYBR Green I-labelled cells. Cells from the virus-free aliquots and from the not-labelled virus-added aliquot were sorted by setting a gate that included the entire population. For virus-added cultures labeled with FM 1-43, the sorting gates included cells with either reduced or normal orange fluorescence, compared to the virus-free culture. In the case of virus-added cultures labeled with CM-H_2_-DCFDA or SYBR Green I, we set a double-gate criterion for cells with increased green fluorescence and relatively low red fluorescence and side scatter signal. Cells were deposited into 384-well plates containing 0.6 µL per well of 1× TE buffer (10 mM Tris-HCl, 1 mM EDTA, pH 8.0) and stored at −80°C until further processing. Sorted plates included single cells, blanks (no doplet deposition), and positive sorting controls (multiple cells into one well).

### MDA reaction

Sorted cells were lysed and their DNA was denatured using cold KOH [25]. The genomic DNA was amplified using real-time multiple displacement amplification (MDA) [25], [26] in 10 µL final volume reactions. The MDA reactions contained 2 U/uL Repliphi polymerase (Epicentre Biotechnologies, Madison, WI, USA), 1x reaction buffer (Epicentre Biotechnologies, Madison, WI, USA), 0.4 mM each dNTP (Epicentre Biotechnologies, Madison, WI, USA), 2 mM DTT (Epicentre Biotechnologies, Madison, WI, USA), 50 mM phosphorylated random hexamers (IDT) and 1 µM SYTO-9 (Invitrogen Co., Carlsbad, CA, USA). The MDA reactions were conducted at 30°C for 16 h followed by a polymerase denaturation step at 65°C for 15 min. Successful reactions were determined based on the real-time kinetics (increase in SYTO-9 fluorescence signal) and the melting curves measured with a FLUOstar Omega (BMG) plate reader. Time for amplification was determined using an in-house algorithm developed to calculate the critical amplification point (Cp), described as the time necessary to reach half of the maximum accumulated fluorescence for each sample. The Cp is inversely correlated to the amount of DNA template [27]. The amplified genomic DNA was stored at −80°C until further use for PCR screening.

### PCR screening of MDA products

The MDA products were diluted 50-fold in sterile TE buffer and 1 µl or 0.5 µL aliquots of the dilute products served as template in 50 µl standard or 5 µL real-time PCR reactions, respectively. Previously described primers (**Table S1**) were used to amplify genes encoding the Major Capsid Protein (MCP) in EhVs [12], a calcium binding protein (GPA) in *E. huxleyi* [13], universal eukaryotic 18S rRNA (primers Euk1A [28] and Euk516R [29]) and prokaryotic and plastid 16S rRNA (primers 27F and 1492R) [30]. The PCR reactions contained 1U Taq DNA polymerase (Promega), 1 × PCR reaction buffer (Promega), 0.25 mM dNTPs, 1.5–2.5 mM MgCl_2_ and 10 pmol of each primer. PCR products from standard reactions were resolved by standard gel electrophoresis, labeled with GelRed™ DNA label (1–2×10^4^ dilution of commercial stock) (Phenix Research, Candler, NC, USA). Reaction kinetics and amplicon melting curves served as proxies detecting amplification of target genes in real-time PCRs. Standard PCRs were performed in an iCycler thermal cycler (Life Science Research, Hercules, CA, USA). Real-time PCRs were performed using LightCycler 480 SYBR Green I Master mix (Roche Applied Science, Indianapolis, IN, USA) in a LightCycler® 480 II real time thermal cycler (Roche Applied Science, Indianapolis, IN, USA). Single cell sorting, whole genome amplification and real-time PCR screens were performed at the Bigelow Laboratory Single Cell Genomics Center (www.bigelow.org/scgc).

## Supporting Information

Figure S1 Representative biparametric flow cytometry plots showing a postinfection time series of *E. huxleyi* cells labeled with the lipid-specific fluorescence dye FM 1-43, (A) non-inoculated (virus-free) control culture, all cells are in a single cluster (red) with acquired high-orange fluorescence and (B) culture inoculated with EhV-86 viruses (virus-added). A cell subpopulation with low-orange fluorescence (green cluster) developed in time in the virus-added culture. Infected and non-infected cells were discriminated on the basis of their red autofluorescence (610 nm) versus orange dye fluorescence (488 nm). Cells for multiple displacement amplification (MDA) and downstream PCR amplification were sorted 20 h post-inoculation from both green and red subpopulations.
(TIF)

Figure S2 Representative biparametric flow cytometry plots showing a post-infection time series of *E. huxleyi* cells labeled with CM-H2DCFDA fluorescence dye. **(A)** non-inoculated (virus-free) control culture and **(B)** culture inoculated with EhV-86 viruses (virus-added). Cells were discriminated on the basis of their red autofluorescence (610 nm) or green dye fluorescence (522 nm) signals versus side scatter signal. The virus-free culture showed an increasing cell subpopulation with high-green fluorescence and relatively higher red fluorescence and side scatter signals (marked by squares) throughout the 48 h period of study, probably as a result of the accumulation of intracellular ROS in some cells due to normal cellular metabolism. From the 20 h post-inoculation sampling point onwards the virus-added culture also showed a cell subpopulation with high-green fluorescence (lower than that in the virus-free culture), however, these cells had relatively lower red fluorescence and side scatter signals (marked by circles).
(TIF)

Figure S3 Representative biparametric flow cytometry plots showing a post-infection time series of *E. huxleyi* cells labeled with SYBR Green I fluorescence dye. **(A)** non-inoculated (virus-free) control culture and **(B)** culture inoculated with EhV-86 viruses (virus-added). Cells were discriminated on the basis of their red autofluorescence (610 nm) versus side scatter or green dye fluorescence (522 nm) signals. Both cultures showed a distinctive cell subpopulation with increased green fluorescence signal (green cluster) from at least 6 h post-inoculation, probably because of the presence of dividing cells or diploid cells in the culture, with relatively higher DNA content. However, from the 20 h post-inoculation onwards the higher green fluorescence group differed between the virus-free and the virus-added cultures with respect to the cells' red fluorescence and side scatter signals, which were relatively high in the virus-free culture (marked by squares) but low in the virus-added culture (marked by ovals).
(TIF)

Figure S4 Representative biparametric flow cytometry plots showing a post-infection time series of *E. huxleyi* cells without addition of any fluorescence dye. **(A)** non-inoculated (virus-free) control culture and **(B)** culture inoculated with EhV-86 viruses (virus-added). Cells were discriminated on the basis of their red autofluorescence (610 nm) or green fluorescence (522 nm) signals versus side scatter signal. In the absence of fluorescence dyes the cells' red autofluorescence and green fluorescence levels did not change throughout the 48 h sampling period, both in the virus-free and the virus-added cultures.
(TIF)

Figure S5 **(A)** Critical point (Cp) distribution for whole genome multiple displacement amplification on a microplate containing sorted *E. huxleyi* strain CCMP 1516 cells labeled with the lipid-specific FM 1-43 dye. Mean Cp is indicated for each group. **(B)** Example of kinetics curve in a single sorted cell well.
(TIF)

Table S1 List of PCR primers, and their sequences, used in this study. F and R denote forward and reverse primer respectively.
(DOC)

File S1 Suitability of the stained and sorted cells for MDA and PCR screening.
(DOC)
