# Supplementary figures and images for: Targeted Sorting of Single Virus-Infected Cells of the Coccolithophore Emiliania huxleyi
Source: PLoS One. 2011 Jul 26;6(7):e22520. doi: 10.1371/journal.pone.0022520 (PMC3144233; doi:10.1371/journal.pone.0022520)

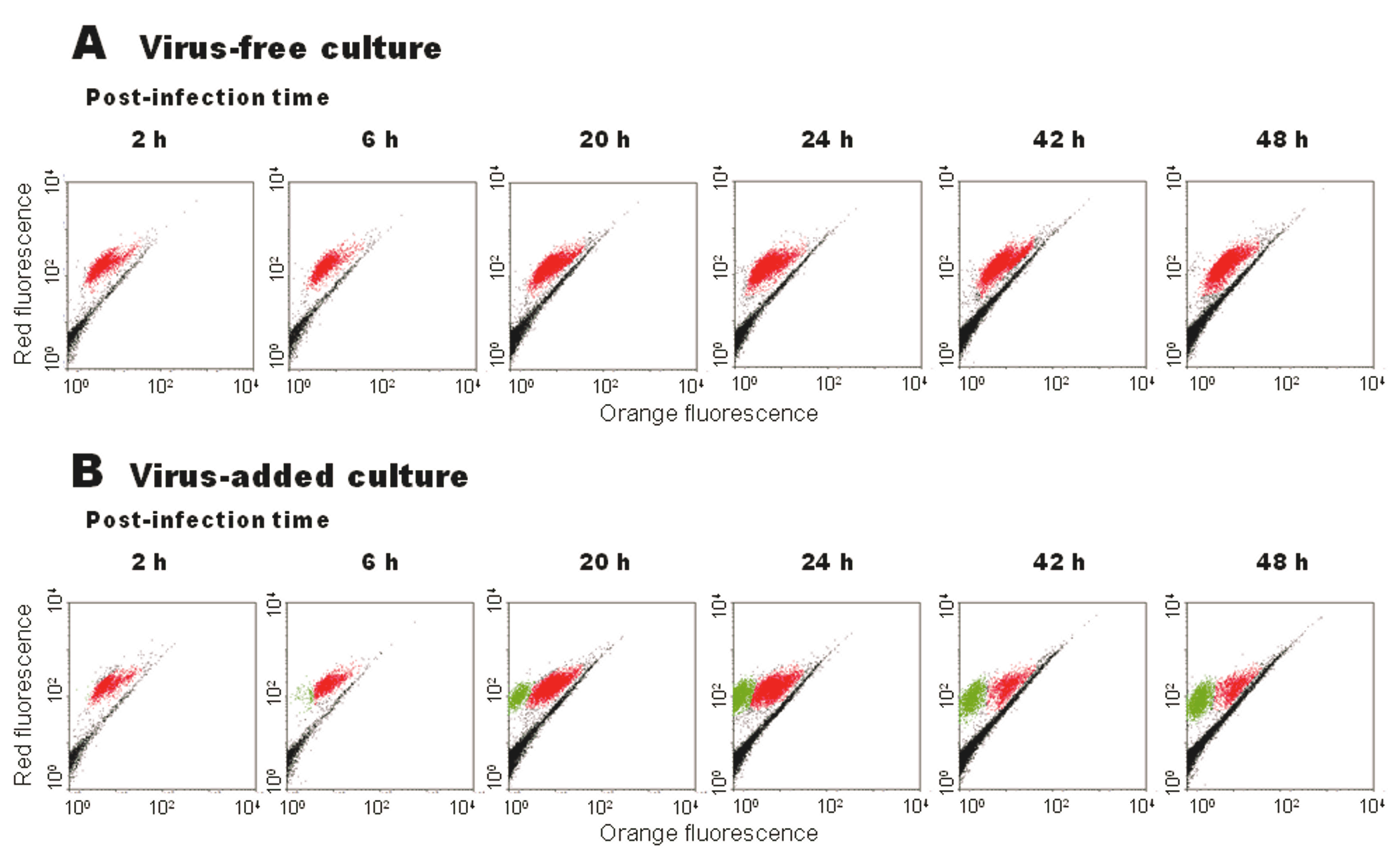

Supplement: Figure S1 — Representative biparametric flow cytometry plots showing a postinfection time series of E. huxleyi cells labeled with the lipid-specific fluorescence dye FM 1-43, (A) non-inoculated (virus-free) control culture, all cells are in a single cluster (red) with acquired high-orange fluorescence and (B) culture inoculated with EhV-86 viruses (virus-added). A cell subpopulation with low-orange fluorescence (green cluster) developed in time in the virus-added culture. Infected and non-infected cells were discriminated on the basis of their red autofluorescence (610 nm) versus orange dye fluorescence (488 nm). Cells for multiple displacement amplification (MDA) and downstream PCR amplification were sorted 20 h post-inoculation from both green and red subpopulations. (TIF) [file pone.0022520.s001.tif]

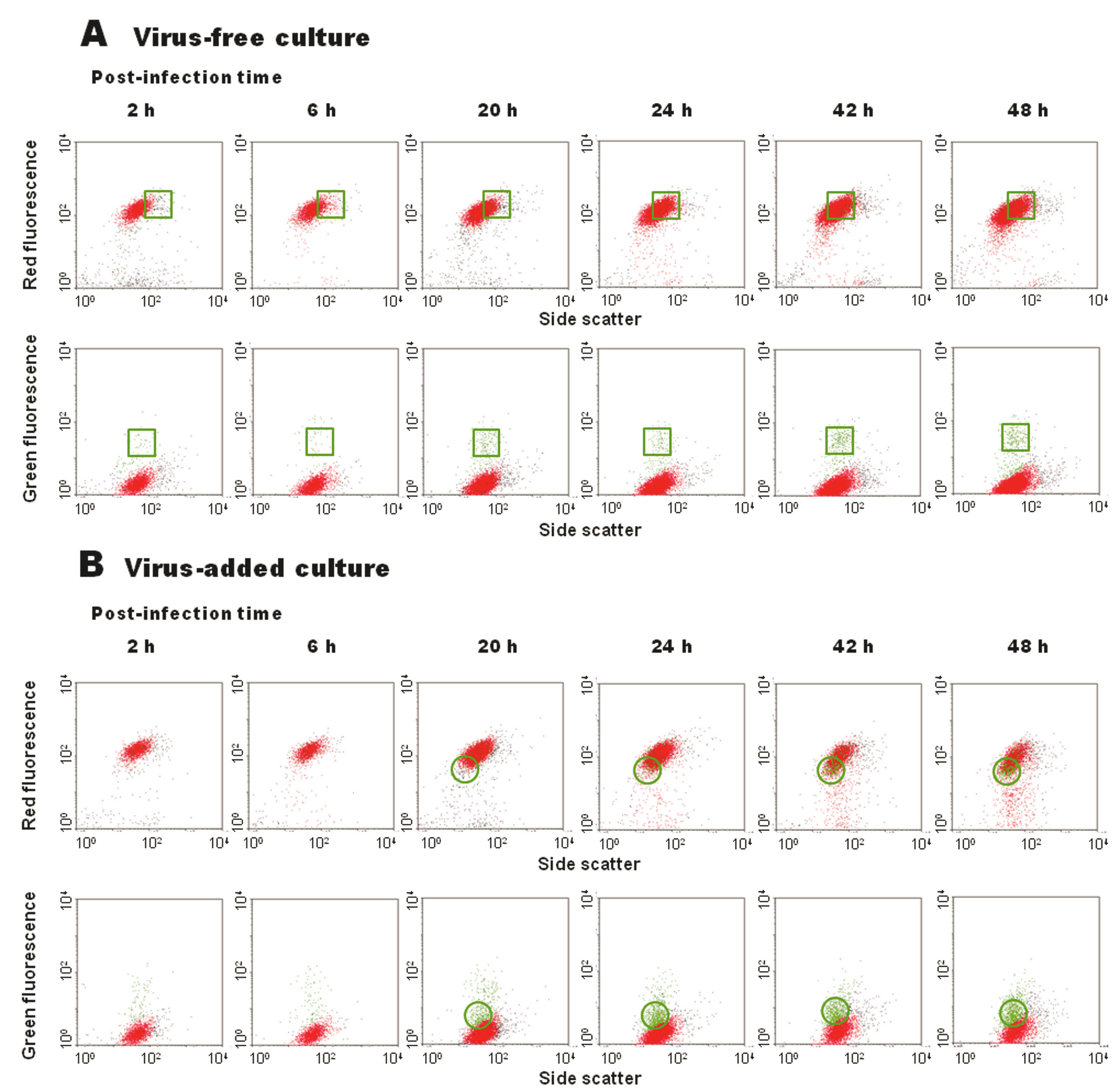

Supplement: Figure S2 — Representative biparametric flow cytometry plots showing a post-infection time series of E. huxleyi cells labeled with CM-H2DCFDA fluorescence dye. (A) non-inoculated (virus-free) control culture and (B) culture inoculated with EhV-86 viruses (virus-added). Cells were discriminated on the basis of their red autofluorescence (610 nm) or green dye fluorescence (522 nm) signals versus side scatter signal. The virus-free culture showed an increasing cell subpopulation with high-green fluorescence and relatively higher red fluorescence and side scatter signals (marked by squares) throughout the 48 h period of study, probably as a result of the accumulation of intracellular ROS in some cells due to normal cellular metabolism. From the 20 h post-inoculation sampling point onwards the virus-added culture also showed a cell subpopulation with high-green fluorescence (lower than that in the virus-free culture), however, these cells had relatively lower red fluorescence and side scatter signals (marked by circles). (TIF) [file pone.0022520.s002.tif]

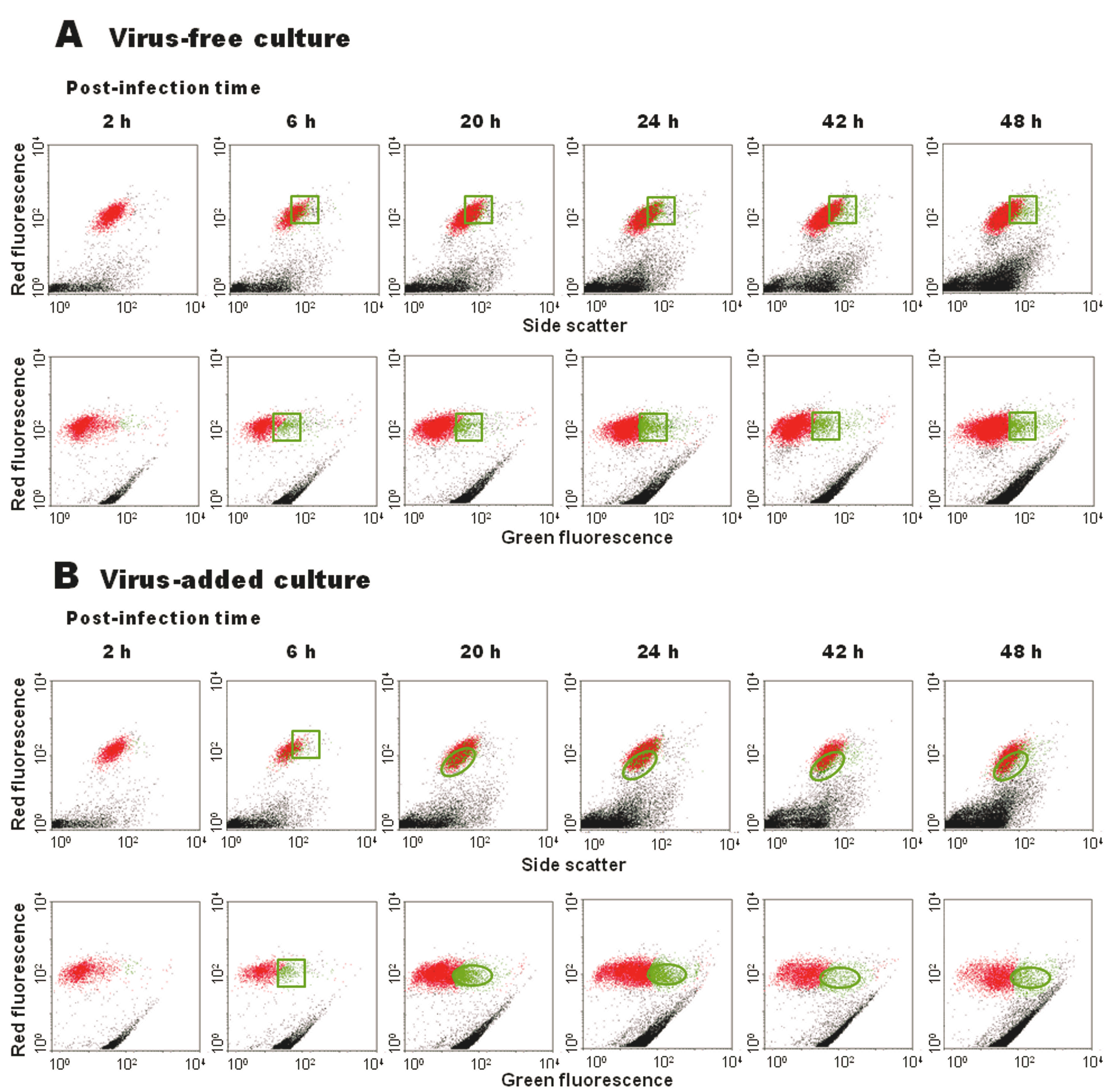

Supplement: Figure S3 — Representative biparametric flow cytometry plots showing a post-infection time series of E. huxleyi cells labeled with SYBR Green I fluorescence dye. (A) non-inoculated (virus-free) control culture and (B) culture inoculated with EhV-86 viruses (virus-added). Cells were discriminated on the basis of their red autofluorescence (610 nm) versus side scatter or green dye fluorescence (522 nm) signals. Both cultures showed a distinctive cell subpopulation with increased green fluorescence signal (green cluster) from at least 6 h post-inoculation, probably because of the presence of dividing cells or diploid cells in the culture, with relatively higher DNA content. However, from the 20 h post-inoculation onwards the higher green fluorescence group differed between the virus-free and the virus-added cultures with respect to the cells' red fluorescence and side scatter signals, which were relatively high in the virus-free culture (marked by squares) but low in the virus-added culture (marked by ovals). (TIF) [file pone.0022520.s003.tif]

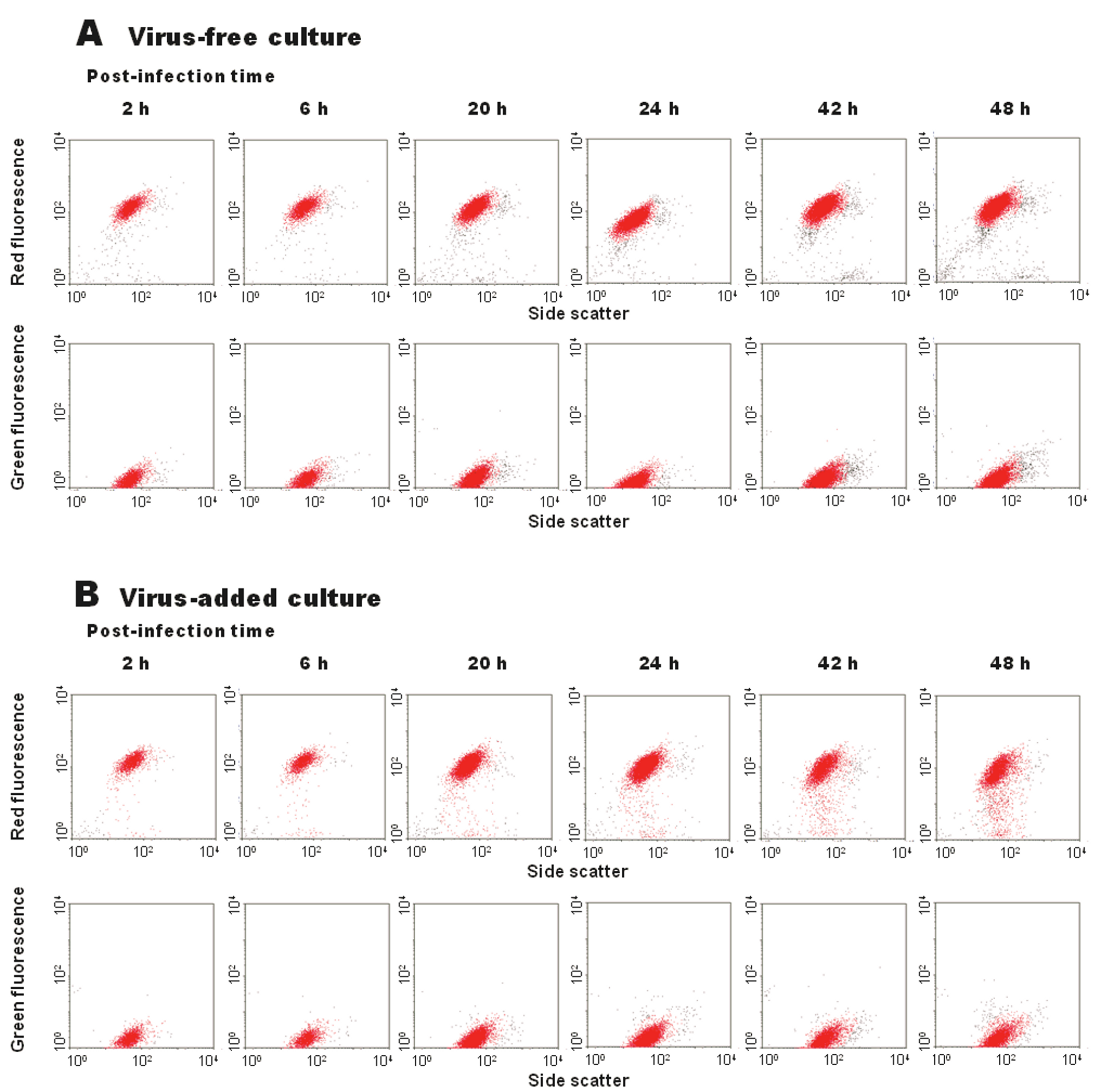

Supplement: Figure S4 — Representative biparametric flow cytometry plots showing a post-infection time series of E. huxleyi cells without addition of any fluorescence dye. (A) non-inoculated (virus-free) control culture and (B) culture inoculated with EhV-86 viruses (virus-added). Cells were discriminated on the basis of their red autofluorescence (610 nm) or green fluorescence (522 nm) signals versus side scatter signal. In the absence of fluorescence dyes the cells' red autofluorescence and green fluorescence levels did not change throughout the 48 h sampling period, both in the virus-free and the virus-added cultures. (TIF) [file pone.0022520.s004.tif]

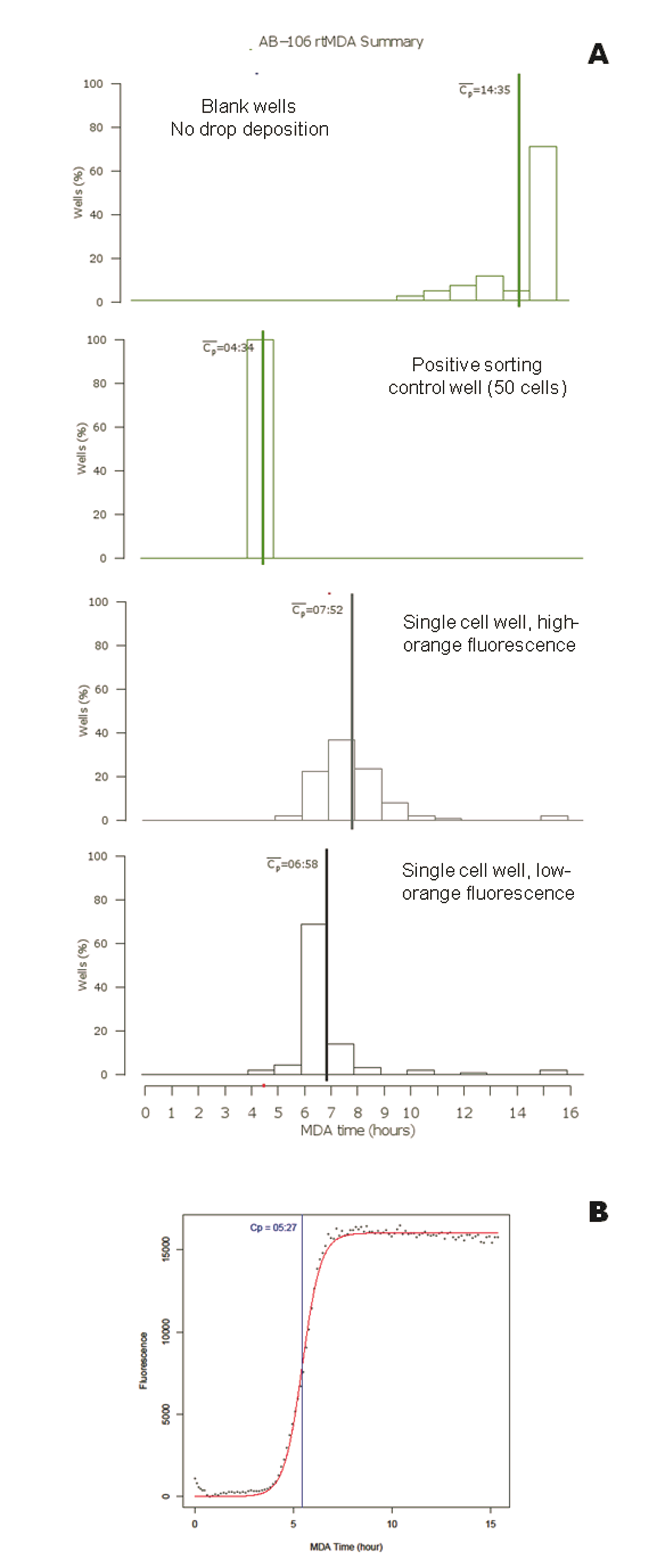

Supplement: Figure S5 — (A) Critical point (Cp) distribution for whole genome multiple displacement amplification on a microplate containing sorted E. huxleyi strain CCMP 1516 cells labeled with the lipid-specific FM 1-43 dye. Mean Cp is indicated for each group. (B) Example of kinetics curve in a single sorted cell well. (TIF) [file pone.0022520.s005.tif]
