# Supplementary material for: Targeted Sorting of Single Virus-Infected Cells of the Coccolithophore Emiliania huxleyi
Source: PLoS One. 2011 Jul 26;6(7):e22520. doi: 10.1371/journal.pone.0022520 (PMC3144233; doi:10.1371/journal.pone.0022520)
Supplement: Table S1 — List of PCR primers, and their sequences, used in this study. F and R denote forward and reverse primer respectively. (DOC) [file pone.0022520.s006.doc]

**Table S1.** List of PCR primers, and their sequences, used in this study. F and R denote forward and reverse primer respectively.

| Primer | Sequence |
| --- | --- |
|  |  |
| GPA-F1[1] | 5’-GAG GAG GAG AAG CCG AGC CT-3’ |
| GPA-R1[1] | 5’-CTT GAA TCC TCT GTG CTG AGC GAG-3’ |
| MCP-F1[2] | 5’-GTC TTC GTA CCA GAA GCA CTC GCT-3’ |
| MCP-R1[2] | 5’-ACG CCT CGG TGT ACG CAC CCT CA-3’ |
| Euk1A[3] | 5’-CTG GTT GAT CCT GCC AG-3’ |
| Euk516R[4] | 5’-ACC AGA CTT GCC CTC C-3’ |
| 27F[5] | 5'-AGAGTTT GATCMTGGCTCAG-3' |
| 1492R[5] | 5'-TACGGYTACCTTGTTAC GACTT-3' |

1. Schroeder DC, Biggi GF, Hall M, Davy J, Martínez Martínez J, et al. (2005) A genetic marker to separate *Emiliania huxleyi* (Prymnesiophyceae) morphotypes. Journal of Phycology 41: 874-879.

2. Schroeder DC, Oke J, Malin G, Wilson WH (2002) Coccolithovirus (Phycodnaviridae): Characterisation of a new large dsDNA algal virus that infects *Emiliania huxleyi*. Archives of Virology 147: 1685-1698.

3. Sogin ML, Gunderson JH (1987) Structural diversity of eukaryotic small subunit ribosomalRNAs. Evolutionary implications. Annales of the New York Academy of Science 503: 125-139.

4. Amann RI, Binder BJ, Olson RJ, Chisholm SW, Devereux R, et al. (1990) Combination of 16S rRNA-targeted oligonucleotide probes with flow cytometry for analyzing mixed microbial populations. Applied and Environmental Microbiology 56: 1919-1925.

5. Lane DJ (1991) 16S/23S rRNA sequencing. In: Stackebrandt E, Goodfellow M, editors. Nucleic acid techniques in bacterial systematics. Chichester: Wiley. pp. 115-175.
