# Supplementary material for: Targeted Sorting of Single Virus-Infected Cells of the Coccolithophore Emiliania huxleyi
Source: PLoS One. 2011 Jul 26;6(7):e22520. doi: 10.1371/journal.pone.0022520 (PMC3144233; doi:10.1371/journal.pone.0022520)

**File S1 – Suitability of the stained and sorted cells for MDA and PCR screening –** An *E. huxleyi* strain CCMP 1516 culture (approx. 1.4 × 10^5^ cells ml^-1^) was split into two aliquots and one was inoculated with EhV-86 (30:1 virus:host ratio). At 20 h post-inoculation three 1 ml aliquots from both cultures were labeled with CM-H_2_DCFDA, SYBR Green I or FM 1-43. A fourth aliquot from each culture was taken but no fluorescence dye was added to it. Cells were sorted from all the aliquots and deposited separately into the wells of a 384-well plate. Cells from the virus-free culture and the not-stained virus-added culture were sorted by setting a gate that included the entire population. For virus-added cultures stained with CM-H_2_-DCFDA or SYBR Green I we set a double-gate criterion for cells with increased green fluorescence and relatively low red fluorescence and side scatter signal (**Figures S2-S3**). In the case of virus-added cultures stained with FM 1-43 the sorting gate included cells with reduced orange fluorescence, compared to the virus-free culture (**Figure S1**). Of the 384 wells, 336 were dedicated for single cells, 40 were used as blanks and 8 were positive sorting controls (100 cells from the virus-free culture or 50 cells from the virus-added culture per well). Additionally, the four wells in the top left-hand side and the four wells in the top right-hand side of the plate received each the addition of 1 ng of human gDNA, after sorting (positive MDA controls). Horizontally, the plate was divided into two; the top half contained cells sorted from the virus-free culture and the bottom half from the virus-added culture. Vertically, the plate was divided into four sections, each dedicated to either not stained, SYBR Green I-stained, CM-H_2_DCFDA-stained or FM 1-43-stained cells.

MDA was performed on all 384 wells. Lack of amplification on the blank wells after 16 h and amplification within the first four hours in multiple-cell wells and those containing human gDNA showed the overall effectiveness of the sorting process, the suitability of the MDA reaction conditions and the lack of DNA contamination in the blanks and the MDA reagents (data not shown). MDA results from single sorts for this experiment are summarized in **File S1- Table A (below)**.

Randomly selected MDA products (11 from the top half and 12 from the bottom half of the plate) including blanks, multiple-cell sorts and single-cell sorts were screened by PCR using *E. huxleyi*-specific GPA primers^1^, eukaryotic 18S rRNA primers (Euk1A and Euk516R)^2,3^ and 16S rRNA primers (27F and 1492R)^4^, the latter was used to check for bacterial contamination. The sequences for these primers are listed in **Table S1**. Those same 23 MDA products and an additional 34 products from the bottom half of the plate (i.e. sorts from the virus-added culture) were also screened by PCR using the EhV-specific primers MCP^5^. The 18S rRNA primers produced amplicons on more of the selected MDA products than the GPA primers. The GPA-PCR reaction was generally more successful on MDA products from virus-infected cells. Every sample that was screened with the 16S rRNA primers produced an amplicon, yet direct sequencing revealed that the amplified fragments corresponded unequivocally to *E. huxleyi*’s plastids and not bacterial contamination (data not shown). As expected, the MCP-PCR reaction did not produce amplicons from any of the virus-free culture MDA products (data not shown) but it produced the correct amplicon from all the virus-added MDA products with Cp < 12 h. None of the products from sorting blanks and failed MDA reactions yielded an amplicon for any of the primer sets, as was true for most of the MDA products with Cp ≥ 12 h (only two exceptions, with MCP primers) (**File S1- Figure A - below)**.

**References**

1. D.C. Schroeder, G.F. Biggi, M. Hall et al., *J. Phycol.* **41**, 874 (2005).

2. M. L. Sogin and J. H. Gunderson, *Annals of the New York Academy of Sciences* **503**, 125 (1987).

3. R. I. Amann, B. J. Binder, R. J. Olson et al., *Appl. Environ. Microbiol.* **56** (6), 1919 (1990).

4. D. J. Lane, in *Nucleic acid techniques in bacterial systematics*, edited by E. Stackebrandt and M. Goodfellow (Wiley, Chichester, 1991), pp. 115.

5. D. C. Schroeder, J. Oke, G. Malin et al., *Arch. Virol.* **147** (9), 1685 (2002).

**File S1- Table A** Summary of MDA results for single sorted cells with and without addition of fluorescent dyes. Cells from the virus-free culture and the not-stained virus-added culture were sorted by setting a gate that included the entire population. For virus-added cultures incubated with CM-H_2_-DCFDA or SYBR Green I we set a double-gate criterion for cells with increased green fluorescence and relatively low red fluorescence and side scatter signal. For virus-added cultures incubated with FM 1-43 the sorting gate included cells with reduced orange fluorescence, compared to the virus-free culture.

| Culture treatment | Fluorescence dye | MDA^a^ | | |
| --- | --- | --- | --- | --- |
|  |  | Cp <8 | 12> Cp ≥8 | Cp ≥12 |
| Virus-free | Not-stained | 45% | 20% | 35% |
|  | FM 1-43 | 57.5% | 7.5% | 35% |
|  | CM-H_2_DCFDA | 72% | 12% | 16% |
|  | SYBR GreenI | 45% | 20% | 35% |
| Virus-added | Not-stained | 87.5% | 2.5% | 9% |
|  | FM 1-43 | 69% | 19% | 12% |
|  | CM-H_2_DCFDA | 66.5% | 12% | 21.5% |
|  | SYBR GreenI | 74% | 9.5% | 16.5% |

^a^ Results are presented as percentage of wells containing single-sorted cells. Time for amplification was determined from the critical amplification point (Cp, in hours), described as the time necessary to reach half of the maximum accumulated fluorescence for each sample.

**File S1- Figure A.** Gel electrophoresis of PCR fragments amplified from MDA products using MCP gene primers (specific to EhVs), GPA gene primers (specific to *E. huxleyi*) and 18S rRNA primers (specific to eukaryotic cells). Numbers indicate the number of cells sorted and subsequently used for MDA amplification. **↓MDA** indicates that the PCR template was from a failed MDA reaction (no accumulated fluorescence). Different colours denote not stained (white), SYBR Green I-stained (green), CM-H_2_DCFDA-stained (yellow) and FM 1-43-stained (orange) cells. * indicates MDA reactions with Cp ≥ 12 hours Positive and negative PCR amplification controls are marked **+** and **-**. Lanes M1, M2 and M3 correspond to DNA molecular markers 100 bp (NEB), 100-1500 bp (LONZA) and 1 kbp (NEB), respectively.


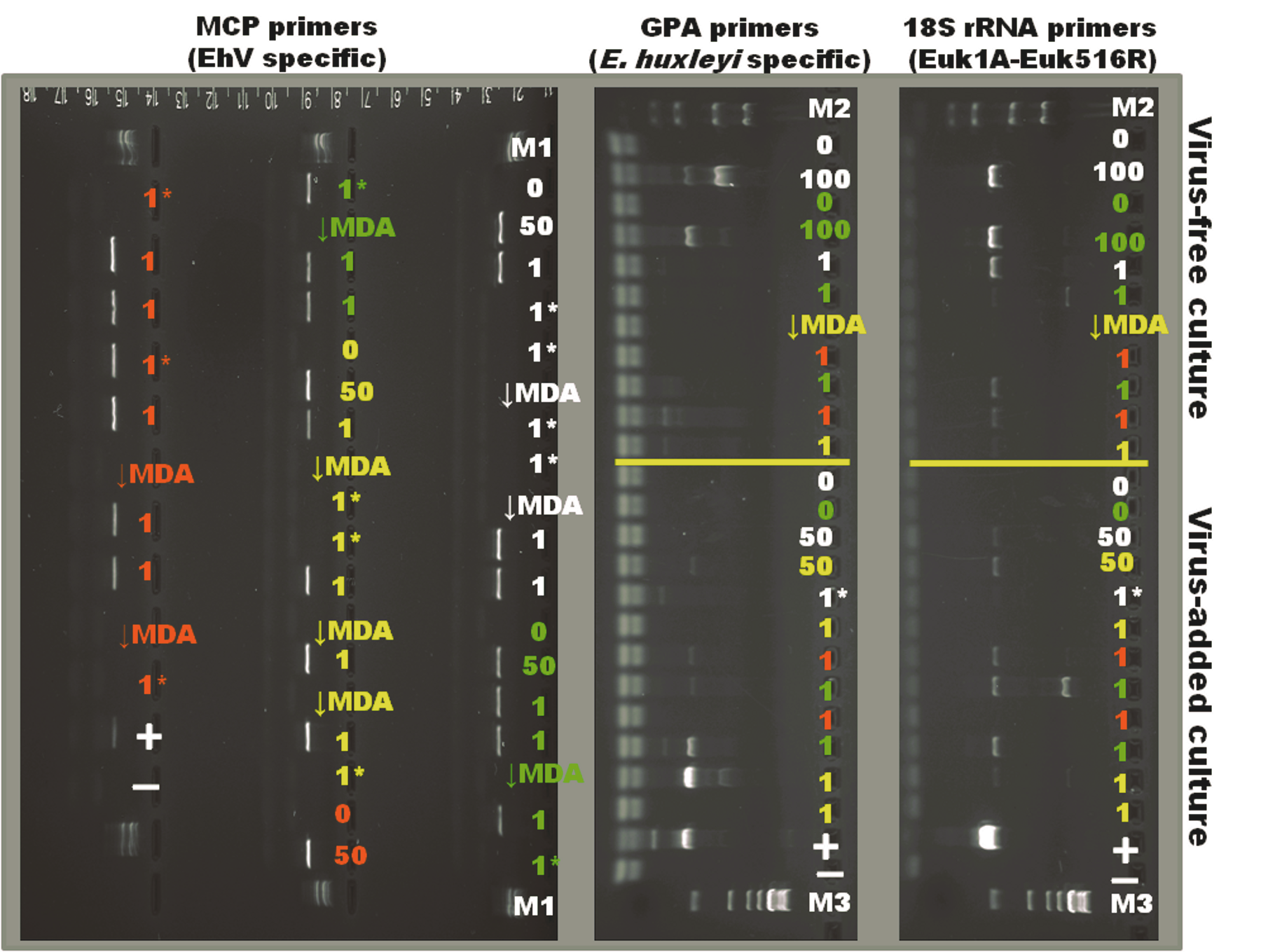

Supplement: File S1 — Suitability of the stained and sorted cells for MDA and PCR screening. (DOC) [file pone.0022520.s007.doc]
